# Surgical Management of Unstable Trochanteric Fractures of the Femur Using Cemented Hemi-Prosthesis in Elderly Patients: A Prospective Study

**DOI:** 10.7759/cureus.45351

**Published:** 2023-09-16

**Authors:** Vilasagarapu Trilok, Ranganatha Babu Kurupati, Nagesh Sherikar, Moinuddin Basha K, Rakshith Chakravarthy H Y

**Affiliations:** 1 Orthopaedics and Trauma, Sri Bhavani Hospital, Vijayawada, IND; 2 Orthopaedics and Trauma, MVJ Medical College and Research Hospital, Bangalore, IND

**Keywords:** cemented, elderly, comminuted, hhs, intertrochanteric

## Abstract

Background

In the case of elderly patients suffering from osteoporosis, the primary objectives of addressing comminuted intertrochanteric fractures are centered upon the recuperation of the patients’ pre-fracture levels of activity, the expeditious promotion of full weight-bearing capacity, and the minimization of the likelihood of further surgical interventions. The adoption of hemiarthroplasty as a method for comminuted intertrochanteric fractures is proven as a means of hastening the recovery process, enabling early weight-bearing and mitigating the problems associated with extended bed rest. The outcomes that resulted from the application of this technique will be evaluated and analyzed as part of this study’s objectives.

Methodology

A prospective study was conducted over the course of one year at a tertiary care hospital in the northern part of India. The study comprised a total of 30 individuals; however, unfortunately, one of the patients could not be located for further analysis. Patients of either gender in the age group of over 60 years old and with unstable osteoporotic intertrochanteric fractures were included (AO Foundation/Orthopaedic Trauma Association type 31-A2.2, A2.3, or 31-A3 group). Patients were observed at one, three, and six months after the surgical operation. The Harris Hip Score (HHS) was used for the functional outcome evaluation.

Results

Throughout the course of our analysis, we saw an increase in the overall HHS that was statistically significant. The HHS exhibited a mean value of 34.33 during the period of discharge, with a range of 32 to 39. It increased to 55.34 (range = 52-59) after one month of follow-up, and it continued to rise to 85.03 (range = 63-89) after three months of follow-up. It is important to note that the mean HHS reached 95.24 (range = 63-98) by the sixth month of follow-up. The study showed a statistically significant upward trend in HHS scores across all time periods (p < 0.001).

Conclusions

Early postoperative ambulation was made possible with the use of cemented prostheses, which contributed to patients’ overall improvements in their functional results. Cemented primary bipolar hemiarthroplasty has emerged as a promising alternative for the treatment of unstable intertrochanteric fractures. The enhanced functional outcomes measured by the HHS provide evidence of this. The transtrochanteric technique has shown advantages in retaining the anatomical integrity of external rotators, minimizing the necessity for their resection, and reducing the danger of sciatic nerve injury. These advantages were displayed by the transtrochanteric approach. Moreover, owing to the implementation of wiring techniques, the larger trochanter could be conserved, resulting in enhanced postoperative recovery and expediting the return to the preoperative condition. When compared with other techniques of internal fixation, the utilization of cemented bipolar hemiarthroplasty demonstrated much-reduced rates of complications, such as the need for further surgery and implant failure.

## Introduction

The intertrochanteric (IT) fracture is one of the most frequent types of hip fractures, and it is more frequent in older adults with osteoporotic bones. These fractures are typically brought on by low-energy trauma, such as trivial falls. The incidence of osteoporosis among the elderly is a key factor in the growth of IT fractures [1]. IT fractures account for around 45% of all hip fractures, and 35-40% of these fractures are unstable, which necessitate surgery, and have high rates of morbidity and mortality [2].

IT fractures are categorized into two groups, namely, stable and unstable, based on the involvement of the lesser trochanter, the presence of a reverse fracture line, the degree of posteromedial comminution, the shattering of the greater trochanter, and the occurrence of a lateral cortical breach [3].

Fractures of the trochanter that are unstable and extensively comminuted are difficult to repair in older patients who also have osteoporosis. Failure rates for the dynamic hip screw range from 5% to 12% [4] because weight bearing is limited by the dynamic hip screw. Extreme collapse and the removal of the lag screw are all associated with internal fixation [5].

It has been demonstrated that proximal femoral nails and other intramedullary interlocking devices improve outcomes in unstable IT fractures and have a lower tendency to cause cut-outs in osteoporotic bone. Particularly for unstable fracture types, head perforations and excessive sliding resulting in shortening continue to be an issue. In internal fixation, a period of restricted mobilization is suggested, which may lead to complications such as atelectasis, bed sores, pneumonia, and deep vein thrombosis. Despite advancements in internal fixing techniques, the proximal femoral nail has a failure rate of 7.1-12.5% in unstable fractures [6].

The goals of treatment for fractures in elderly patients with osteoporosis encompass facilitating the prompt resumption of full weight bearing, reinstating the patient to their pre-injury activity level, and circumventing the need for additional surgical interventions [7].

Comminuted IT fractures have been treated by hemiarthroplasty [8]. The goal of this treatment is to get these patients up and moving as soon as possible, to get them to bear their own weight as soon as possible, and to prevent any complications that may come from prolonged periods of lying down.

Primary hemiarthroplasty for osteoporotic unstable intertrochanteric fractures yielded favorable results in a study by Surapaneni and colleagues [9]. The technique also facilitated quicker mobilization, a speedier recovery to pre-injury levels, and an enhanced quality of life.

The purpose of this study is to evaluate the efficacy of cemented hemiprostheses in treating elderly osteoporosis patients’ unstable intertrochanteric fractures.

## Materials and methods

This study was conducted over the course of one year at a hospital that provided tertiary treatment in the northern part of India. The study comprised a total of 30 individuals; however, unfortunately, one patient could not be located for further analysis. We included patients older than 60 years old who had unstable osteoporotic intertrochanteric fractures (AO Foundation/Orthopaedic Trauma Association (AO/OTA) type 31-A2.2, A2.3, or 31-A3 group). In this study, we included individuals who had suffered from multiple injuries with an absence of any additional bone fractures in the ipsilateral limb. We excluded patients who were unable to undergo surgery due to pathological fractures.

Following the acquisition of informed and written consent from the patients, the surgical procedures were performed using a transtrochanteric technique. A single, highly experienced chief surgeon conducted the procedures on each patient.

Surgical procedure

The patient was positioned in the lateral orientation for the duration of the surgical operation. An incision was created on the side of the hip, focused on the greater trochanter’s proximal part. This was done to access the hip joint. The portion of the incision that was closer to the patient’s body curled backward, heading in the direction of the posterior superior iliac spine. Alongside the skin incisions, tensor fascia lata was also incised. To reveal the fracture, gluteus maximus fibers were dissected proximally along the incision. The greater trochanter fracture was meticulously dissected, and the fragments were withdrawn all the way to the base of the femur neck.

This transtrochanteric window was utilized to remove the head of the femur together with its connected neck. After the femoral neck and head had been removed, the acetabulum underwent a careful inspection to ensure that it was free of any leftover bone pieces. Following that, the femoral canal was prepared with the help of a reamer and successive broaches. On the lateral face of the proximal femur, two holes were bored about 5 cm below the vastus ridge. Subsequently, a stainless-steel wire was inserted into the medullary canal from the outside through one of the holes and then removed through the other hole.

Before the implantation procedure, anteversion was a major consideration. The long axis of the leg served as a reference point for this. The tension of the soft tissue was carefully considered while determining the length of the implant that would be placed in the femur. The length of the limb was verified, and the results showed that the trial implant remained in place with just a movement of a few millimeters after being put through a range of hip movements. Before cementing either the trial or the final implants, meticulous marking was done to indicate the depth of stem insertion on each of them. The goal was to keep the length of the operated limb comparable to that of the contralateral limb intraoperatively.

The calcar was reconstructed using cement, and once the appropriate-sized implant had been inserted, the hip was reduced. After the greater trochanter was placed in its position, holes were drilled into it and a stainless-steel wire was inserted to hold the halves together. The hip’s abductor mechanism was then assisted back into place by the subsequent tightening of the wire, which increased compression over the fracture site.

Follow up

Following the surgery, regular appointments were made for all patients in the outpatient department at one, three, and six months after the operation. During these appointments, the patients got regular follow-up care. A comprehensive clinical evaluation of the patients was performed on each visit. The Harris Hip Score (HHS) was used to evaluate the functional outcomes.

Statistical analysis

Categorical variables were represented by numbers and percentages (%), whereas continuous variables were described by the mean, standard deviation, and median. To ensure that the data were normally distributed, the Kolmogorov-Smirnov test was performed. A non-parametric test is performed if the normality assumption is rejected. When comparing quantitative variables before and after the intervention, the Wilcoxon rank sum test was utilized when the datasets were not normally distributed. A statistically significant result requires a p-value of less than 0.05. SPSS version 21.0 (IBM Corp., Armonk, NY, USA) was used for the analysis. Microsoft Excel (Microsoft Corp., Redmond, WA, USA) was used to construct a data spreadsheet.

## Results

The observations and analyses of the study were conducted in relation to various factors such as gender (Table 1), age (Table 2), and complications such as infection, wire breakage, and dislocation. Functional outcome as measured by the HHS (Table 3). The final results are presented in Table 4 once the follow-up was completed.

**Table 1:** Sex-wise distribution of patients.

	Frequency	Percentage
Female	19	63.33%
Male	11	36.67%
Total	30	100.00%

**Table 2:** Age at which the fracture occurred.

Age (years)	Number	Percentage (%)
≤70	15	50.0
70–80	13	43.3
80–90	1	3.3
>90	1	3.3
Total	30	100.0

**Table 3:** Comparison of the mean Harris Hip Score at all time points.

	Sample size	Mean ± SD	Median	Minimum–Maximum	Inter quartile range	P-value
Preoperative Harris Hip Score	30	0 ± 0	0	0–0	0–0	
Immediate postoperative Harris Hip Score	30	34.33 ± 1.69	34.5	32–39	33–35	<0.0001
One-month postoperative Harris Hip Score	29	55.34 ± 2.47	56	52–59	53–58	<0.0001
Three-month postoperative Harris Hip Score	29	84.03 ± 5.02	85	63–89	82–88	<0.0001
Six-month postoperative Harris Hip Score	29	95.24 ± 6.67	98	63–98	95–98	<0.0001

**Table 4:** Harris Hip Score outcomes at the final follow-up.

Final outcome	Number	Percentage
Excellent	25	86.20
Good	3	10.35
Fair	0	0
Poor	1	3.45
Total	29	100.0

Complications

One patient developed an infection during the first month of treatment, while another patient experienced a dislocation on the fifth postoperative day.

## Discussion

The findings of our investigation are consistent with the findings of previous studies which reported similar trends in terms of the problems and outcomes that were addressed before.

During our analysis, we found that females had a much greater incidence of fractures than men did, with 19 out of 30 (63.3%) cases, as opposed to 11 (33.7%) cases, respectively. This trend is consistent with the observations made by Sinno et al. [10], who found that out of 48 patients, 34 were females and 14 were men. According to earlier research, the increased occurrence of fractures in females might be related to the somewhat lower bone mineral density in females when compared to males. This finding is consistent with the findings of other studies.

In this study, we discovered that the age range of 60-70 included 50% of all patients, while the age range of 70-80 comprised 43.3% of all patients. In addition, one patient (3.3% of the total population) belonged to the age range of 80 to 90 years old, and another patient (3.3% of the total population) was older than 90 years old. The calculated mean age incidence ranged from 61 to 91 years old, with the mean age being 71.7 years old. This number is comparable to the average age reported in earlier research. For example, according to the findings of the study by Kumar et al. [11], the average age was recorded as 72.4 years, with the age range spanning from 65 to 95. In a similar vein, Ukaj and colleagues [12] reported a mean age of 78.3 ± 5.5 years, with ages ranging from 70 to 92, which corresponds with the patterns that were discovered in our investigation.

The AO/OTA system was used to categorize fractures, with an emphasis on including only those fractures that were judged to be unstable. These unstable fractures were further classified as being of the 31A2 and 31A3 types. In our investigation, there were a total of 26 fractures, of which six were categorized as being of the 31A2 type (20%) and 24 of the 31A3 type (80%). As a result of our investigation into the connection between the different types of fractures and the functional results, we concluded that the different types of fractures did not display any discernible association with the functional outcomes measured by the HHS. The HHS for the two different types of fractures were 93.66 and 97, respectively, at the six-month follow-up. These results are consistent with the findings of past research. The functional result score was reported to be 89 in research that was headed by Ukaj et al. [12], and it largely contained 31A3-type fractures. The score was reported to be within a range of 83 to 95.

Using the HHS rating system to examine the effects of the main bipolar hemiprosthesis for unstable IT fractures in 37 senior patients, 17 instances (45%) were classified as excellent and 14 cases (37%) as good after a 12-month period. Rodoop et al. [4] examined the effectiveness of primary bipolar hemiprostheses in treating unstable IT fractures in a group of 37 elderly patients. Meanwhile, 20 patients aged 65 and up with unstable IT femur fractures were studied by Puttakemparaju et al. [13] who were treated with bipolar hemiarthroplasty. Their evaluation of functional outcomes, which was determined by the HHS, showed a mean score of 78.2 at the conclusion of the first six months, which increased to 83.25 after 24 months. Notably, a favorable functional result was determined to have been achieved based on the HHS at the two-year mark. HHSs of various other studies compared to our study are shown in Table 5.

**Table 5:** Harris Hip Scores of various studies.

Study	Mean Harris Hip Score
Patil et al. [14]	80.76 (60–88)
Surapaneni et al. [9]	84.8 (58–97)
Singh et al. [15]	78.86 (61.9–87.8)
Our study	95.24 (63–98)

Postoperative rehabilitation is greatly improved due to the repair of the abductor musculature through the utilization of the transtrochanteric technique during surgery. This has led to an increase in the number of patients in our research who had great outcomes. Because of the wiring of the greater trochanter, not only was there an improvement in abductor power and mechanical strength for free walking but there was also a considerable rise in the HHS. The use of a cemented prosthesis contributed to a more successful and expedient return to the preoperative state.

During our analysis, we saw that the mean HHS was steadily improving across all different time points. The mean HHS was 34.33 at the time of discharge, ranging from 32 to 39. It increased to 55.34 (range = 52-59) after one month of follow-up, and it continued to rise to 85.03 (range = 63-89) after three months of follow-up. It is important to note that the mean HHS had reached 95.24 (range = 63-98) by the sixth-month follow-up. With a p-value less than 0.001, the study demonstrated that there is a statistically significant rising trend in HHSs across all time periods.

When we evaluated the patients’ functional outcomes using the criteria established by the HHS, we discovered that 25 (86%) patients achieved outstanding results, three (11%) attained good results, and there were no patients who were classified as having average results. According to this examination, 3% of patients, specifically one patient, demonstrated unsatisfactory results. The results of our investigation were consistent with those discovered in earlier research.

Regarding the intricacy associated with a surface-level infection, we encountered an individual who necessitated the administration of intravenous antibiotics and debridement to control the infection. The implementation of these interventions yielded favorable outcomes. The range of 8-16% that is published in the literature [16] following primary osteosynthesis compares unfavorably to our reoperation rate of 3.45%, which stands out as being significantly lower. In addition, on the fifth postoperative day, one patient fell out of bed, which resulted in dislocation. This problem was managed by closed reduction, which was then followed by a two-week period during which the patient was immobilized. Then, the patient began to gradually regain mobility. Importantly, the complication rate in our research was just 6.8%, and there were no instances of implant failure, implant loosening, or evidence of erosion or calcar protrusion. These findings were in line with those found in prior research that were equivalent to this.

Patil et al. [14] noted that they had incidences of implant loosening, dislocation, and infection; nevertheless, each case was handled adequately. In the study by Sancheti et al. [17], one patient had an infection that was treated with a two-week course of antibiotics, while another patient’s difficulty walking was related to their condition of Alzheimer’s disease and their lack of cooperation. Both of these factors contributed to the patient’s inability to walk.

Limitations

This research was carried out at a single center, and while it does provide some helpful insights, there is a need for more extensive research that takes place across numerous centers. Because the amount of time spent following up with patients was restricted, future research should focus on undertaking longer-term observations. Additionally, the forthcoming investigation must encompass a more extensive and diverse patient cohort, encompassing individuals from various age demographics. It is important to point out that the diagnosis of osteoporosis was made only on the basis of radiological findings obtained from X-rays. On the other hand, a more thorough examination that made use of procedures such as dual X-ray absorptiometry scans would have further improved the study’s level of detail and precision.

## Conclusions

Early postoperative ambulation was made possible with the use of cemented prostheses, which contributed to patients’ overall improvements in their functional results. Cemented primary bipolar hemiarthroplasty has emerged as a promising alternative for the treatment of unstable intertrochanteric fractures. The enhanced functional outcomes measured by the HHS provide evidence of this. The transtrochanteric technique has shown advantages in terms of retaining the anatomical integrity of external rotators, minimizing the necessity for their division, and reducing the danger of sciatic nerve injury. Furthermore, through the implementation of wiring techniques, preservation of the greater trochanter was achieved, resulting in enhanced postoperative recovery and ultimately facilitating a prompt return to the preoperative condition.

## References

[1] Kulkarni G, Limaye R, Kulkarni M, Kulkarni S (2006). Intertrochanteric fractures. Indian Orthop.

[2] Grimsrud C, Monzon RJ, Richman J, Ries MD (2005). Cemented hip arthroplasty with a novel cerclage cable technique for unstable intertrochanteric hip fractures. J Arthroplasty.

[3] Jayapalan J, Pandian P, Pandian S, Rajendiran C, Duraisamy V (2015). Unstable intertrochanteric fracture in elderly treated with cemented bipolar hemiarthroplasty and trochanteric reconstruction. J Evid Based Med Healthc.

[4] Rodop O, Kiral A, Kaplan H, Akmaz I (2002). Primary bipolar hemiprosthesis for unstable intertrochanteric fractures. Int Orthop.

[5] Bannister GC, Gibson AG, Ackroyd CE, Newman JH (1990). The fixation and prognosis of trochanteric fractures. A randomized prospective controlled trial. Clin Orthop Relat Res.

[6] Pal C, Dinkar K, Mittal V, Goyal A, Singh M, Hussain A (2016). Role of bipolar hemiarthroplasty and total hip arthroplasty in unstable intertrochanteric fracture femur. J Orthop Allied Sci.

[7] Elmorsy A, Saied M, Zaied M, Hafez M (2012). Primary bipolar arthroplasty in unstable intertrochanteric fractures in elderly. Open J Orthop.

[8] Haentjens P, Casteleyn PP, De Boeck H, Handelberg F, Opdecam P (1989). Treatment of unstable intertrochanteric and subtrochanteric fractures in elderly patients. Primary bipolar arthroplasty compared with internal fixation. J Bone Joint Surg Am.

[9] Surapaneni SB, Velagapudi NG, Babu Tummala VS (2017). Prosthetic replacement in geriatric intertrochanteric fracture. Int J Orthop Sci.

[10] Sinno K, Sakr M, Girard J, Khatib H (2010). The effectiveness of primary bipolar arthroplasty in treatment of unstable intertrochanteric fractures in elderly patients. N Am J Med Sci.

[11] Kumar Gn K, Meena S, Kumar N V, S M, Raj Mk V (2013). Bipolar hemiarthroplasty in unstable intertrochanteric fractures in elderly: a prospective study. J Clin Diagn Res.

[12] Ukaj S, Gjyshinca B, Podvorica V, Ukaj F, Molliqaj G, Boshnjaku A, Gamulin A (2017). Primary hemiarthroplasty for treatment of unstable pertrochanteric femoral fractures (AO/OTA Type 31 A2.3) in elderly osteoporotic patients. SICOT J.

[13] Puttakemparaju K, Beshaj N (2014). Unstable intertrochanteric fracture in elderly treated with bipolar hemiarthroplasty: a prospective case series. Afr J Trauma.

[14] Patil A, Ansari M, Pathak A, Goregaonkar B, Thakkar CJ (2013). Role of cemented bipolar hemiarthroplasty for communited intertrochanteric fracture femur. IOSR-JDMS.

[15] Singh S, Shrivastava C, Kumar S (2014). Hemi replacement arthroplasty for unstable inter-trochanteric fractures of femur. J Clin Diagn Res.

[16] Leonardsson O, Kärrholm J, Åkesson K, Garellick G, Rogmark C (2012). Higher risk of reoperation for bipolar and uncemented hemiarthroplasty. Acta Orthop.

[17] Sancheti Kh, Sancheti P, Shyam A, Patil S, Dhariwal Q, Joshi R (2010). Primary hemiarthroplasty for unstable osteoporotic intertrochanteric fractures in the elderly: a retrospective case series. Indian J Orthop.

